# Clinical, Virological, Immunological, and Genomic Characterization of Asymptomatic and Symptomatic Cases With SARS-CoV-2 Infection in India

**DOI:** 10.3389/fcimb.2021.725035

**Published:** 2021-12-21

**Authors:** Sanchari Chatterjee, Ankita Datey, Soumya Sengupta, Arup Ghosh, Atimukta Jha, Safal Walia, Sharad Singh, Sandhya Suranjika, Gargee Bhattacharya, Eshna Laha, Supriya Suman Keshry, Amrita Ray, Sweta Smita Pani, Amol Ratnakar Suryawanshi, Rupesh Dash, Shantibhusan Senapati, Tushar K. Beuria, Gulam Hussain Syed, Punit Prasad, Sunil Kumar Raghav, Satish Devadas, Rajeeb K. Swain, Soma Chattopadhyay, Ajay Parida

**Affiliations:** ^1^ Infectious Disease Biology, Institute of Life Sciences, Bhubaneswar, India; ^2^ Infectious Disease Biology, Regional Center for Biotechnology, Faridabad, India

**Keywords:** COVID-19, asymptomatic and symptomatic patients, cytokines, D614G mutation, disease severity, SARS-CoV-2

## Abstract

**Purpose:**

The current global pandemic of coronavirus disease 2019 (COVID-19), caused by severe acute respiratory syndrome coronavirus 2 (SARS-CoV-2), led to the investigation with clinical, biochemical, immunological, and genomic characterization from patients to understand the pathophysiology of viral infection.

**Methods:**

Samples were collected from six asymptomatic and six symptomatic SARS-CoV-2-confirmed hospitalized patients in Bhubaneswar, Odisha, India. Clinical details, biochemical parameters, and treatment regimen were collected from a hospital; viral load was determined by RT-PCR; and the levels of cytokines and circulating antibodies in plasma were assessed by Bio-Plex and isotyping, respectively. In addition, whole-genome sequencing of viral strains and mutational analysis were carried out.

**Results:**

Analysis of the biochemical parameters highlighted the increased levels of C-reactive protein (CRP), lactate dehydrogenase (LDH), serum SGPT, serum SGOT, and ferritin in symptomatic patients. Symptomatic patients were mostly with one or more comorbidities, especially type 2 diabetes (66.6%). The virological estimation revealed that there was no significant difference in viral load of oropharyngeal (OP) samples between the two groups. On the other hand, viral load was higher in plasma and serum samples of symptomatic patients, and they develop sufficient amounts of antibodies (IgG, IgM, and IgA). The levels of seven cytokines (IL-6, IL-1α, IP-10, IL-8, IL-10, IFN-α2, IL-15) were found to be highly elevated in symptomatic patients, while three cytokines (soluble CD40L, GRO, and MDC) were remarkably higher in asymptomatic patients. The whole-genome sequence analysis revealed that the current isolates were clustered with 19B, 20A, and 20B clades; however, 11 additional changes in Orf1ab, spike, Orf3a, Orf8, and nucleocapsid proteins were acquired. The D614G mutation in spike protein is linked with higher virus replication efficiency and severe SARS-CoV-2 infection as three patients had higher viral load, and among them, two patients with this mutation passed away.

**Conclusions:**

This is the first comprehensive study of SARS-CoV-2 patients from India. This will contribute to a better understanding of the pathophysiology of SARS-CoV-2 infection and thereby advance the implementation of effective disease control strategies.

## Introduction

Coronavirus disease 2019 (COVID-19), which was initially reported to cause pneumonia and flu-like illness in the city of Wuhan, China, has now become considerably more perilous, with global efforts undergoing to combat the deadly disease (Yang et al., 2020). The betacoronavirus has a spherical enveloped single-stranded positive sense RNA genome with 5′ cap and 3′ poly-A-tail (Chen et al., 2020).

According to the WHO, 223 countries to date have been affected by the second wave of COVID-19, with 159,319,384 confirmed cases and an increase in the number of deaths around the world. Although the vaccination program in India is at its height, the total number of vaccinations given is 1,77,214,256. The second wave of COVID-19 cases surpassed the 23,340,938 mark and death toll continues to rise beyond 258,317 deaths. As of May 13, 2021, Odisha has 100,313 active cases with 2,304 deaths and 64.5 lakhs vaccinated (Ministry of Health and Human Welfare, GOI).

Previous investigations of severe acute respiratory syndrome coronavirus 1 (SARS-CoV-1) occurrence demonstrated the correlation of disease deterioration and viral load in the nasopharynx (Cheng et al., 2004; Hung et al., 2004). Studies have now differentiated SARS-CoV-1 and SARS-CoV-2 in terms of clinical manifestations (Guan et al., 2020), virus shedding (To et al., 2020), and epidemiological aspects (Wu et al., 2020). However, investigations are required to understand the pathophysiology of SARS-CoV-2, thereby elucidating the association of viremia in different body fluids and disease severity.

Hence, the current investigation was carried out to delineate and compare the clinical manifestations, laboratory findings on viral load, immunological responses, genome sequences, treatment protocol, and its outcome of asymptomatic and symptomatic patients infected with SARS-CoV-2 to understand the pathophysiology of this virus.

## Materials and Methods

### Study Design

In this study, all the patients were admitted to a COVID hospital in Bhubaneswar, Odisha, India, in July 2020, and biological samples including oropharyngeal (OP) swab and blood were collected. The designed study was approved by the institutional ethics committee and the signed consent forms were taken from the concerned patients. The OP swab sample from the patient was tested immediately to detect the presence of SARS-CoV-2 by RT-PCR. Next, OP swabs were collected from the patients in the first (1–3 days), second (5–7 days), and third (8–10 days) phases, while blood samples were collected in one time point (1–5 days).

### Data Collection

The demographic and clinical details of the patients were obtained from the hospital including travel history, treatment details, comorbidities, and symptoms. The data for a few patients were missing due to the absence of tests or delayed results.

### Viral RNA Extraction

Three hundred microliters of OP swab/plasma/serum was taken for viral RNA extraction using TANBead Maelstrom 4800 as per the instructions of the manufacturer. The extracted viral RNA was stored at −80°C until further use.

### qRT-PCR

The qRT-PCR was performed using 5 μl of the extracted RNA from samples using the TRUPCR SARS-CoV-2 RT qPCR Kit V-2.0. The human RNase P served as an internal control, whereas envelope (E) and nucleocapsid (N) genes were targeted for SARS-CoV-2 amplification.

### Viral Copy Number Determination

The viral copy number was determined as described previously (Sanjai Kumar et al., 2021). In brief, the standard curve was generated by using the SARS-CoV-2 N gene. The percentage of copy number/ml was calculated from the corresponding Ct values of all the samples. For obtaining Ct values, cDNA was prepared from the extracted RNA using random hexamers by TaKaRa PrimeScript 1st strand cDNA synthesis kit (Kusatsu). The cDNA was subjected to qPCR (Mesa Green SYBR Green-No ROX, Eurogentec) using nucleocapsid gene-specific primers (FP: GTAACACAAGCTTTCGGCAG and RP: GTGTGACTTCCATGCCAATG).

### ELISA

Plasma samples were used to detect the presence of total antibodies (IgM+IgG+IgA) against SARS-CoV-2 by the COVID-19 (IgM+IgG+IgA) Microlisa kit (J. Mitra & Co. Pvt. Ltd.) as per the protocol of the manufacturer. In brief, 50-µl plasma samples were added to precoated plates and incubated for 30 min at 37°C. Following incubation, the plate was washed with 1× wash buffer five times. One hundred microliters of conjugate solution was added to each well and incubated for 30 min at 37°C. After washing five times, 100 µl of working substrate solution was added and incubated at room temperature for 30 min in the dark. The reaction was stopped using 100 µl of 1 N H_2_SO_4_ and absorbance was measured at 450 nm in a Multiskan reader (Thermo Scientific). The antibody unit was calculated as per the instruction of the manufacturer.

### Bio-Plex

Twenty-five microliters of plasma sample was used to analyze the responses of 38 cytokines and chemokines using the Bio-Plex Human Cytokine/Chemokine Magnetic Bead Panel 96-Well Plate Assay (EMD Millipore) according to the instructions of the manufacturer. The samples were acquired in a Bio-Plex 200 system (Bio-Rad), and cytokine concentrations were calculated using the Bio-Plex manager software with a five-parameter curve-fitting algorithm applied for standard curve calculation.

### Isotyping

To analyze the isotype composition of antibodies in circulation, the plasma of patients was analyzed by the ProcartaPlex Human Antibody Isotyping Panels (Invitrogen, Vienna) according to the instructions of the manufacturer. Briefly, the antibody-coated magnetic bead mixtures were incubated with 25 μl of assay buffer, kit standards, or diluted plasma samples in a ProcartaPlex 96-well plates at room temperature for 1 h. Twenty-five microliters of detection antibodies mixture was then added, and the plates were incubated on an orbital shaker at 600 rpm for 30 min. After that, each well was incubated with 50 μl of diluted streptavidin–phycoerythrin for 30 min. Plates were then washed using a handheld magnetic plate washer. All incubations were performed at room temperature in the dark. Afterwards, the samples were suspended in 120 μl reading buffer. The samples were acquired in a Bio-Plex 200 system (Bio-Rad) and cytokine concentrations were calculated using the Bio-Plex manager software with a five-parameter curve-fitting algorithm applied for standard curve calculation.

### Library Preparation and Whole-Genome Sequencing

The viral amplicon libraries were prepared for whole-genome sequencing using the QIAseq FX DNA Library Kit and QIAseq SARS-CoV-2 Primer Panel as per the instructions of the manufacturer. The samples were pooled and subjected to Illumina NextSeq 550 platform in a 150 × 2 layout, as described before (Raghav et al., 2020).

### Phylogenetic and Mutational Analysis

The mutational and phylogenetic analysis was carried out using the Nextstrain tool as described before (Raghav et al., 2020). Sequences were aligned along with the WH01 reference strain using the Augur wrapper of MAFFT method to carry out mutational analysis, and a phylogenetic tree was constructed using the IQTREE2 tool with 1,000 bootstrap value (Raghav et al., 2020).

### Statistical Analysis

Statistical analysis was performed using the GraphPad Prism software, version 8.0.1. Data were presented as mean ± standard deviation (SD). The non-parametric Mann–Whitney *U* test was used to compare the levels of viral load, cytokines, and antibodies.

## Results

### Clinical and Biological Characteristics of SARS-CoV-2-Infected Asymptomatic and Symptomatic Patients

**Figure 1:**
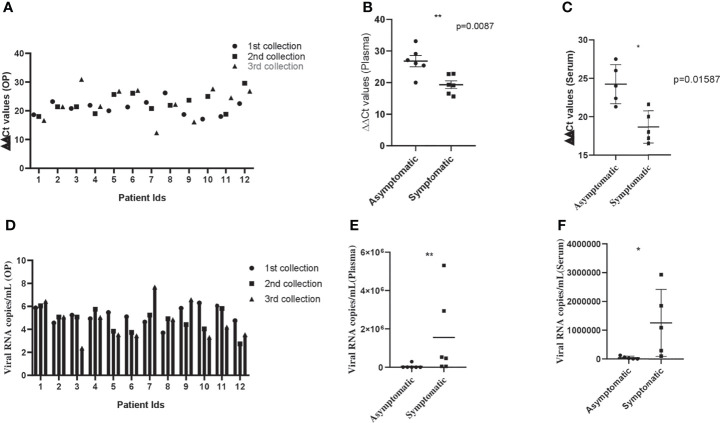
Viral dynamics in patients with asymptomatic and symptomatic disease. **(A)** Bar diagram depicting the ΔΔCT of OP samples collected from patients with asymptomatic and symptomatic COVID-19 at different days of disease onset. **(B, C)** Graph showing ΔΔCT of individual plasma and serum samples collected from asymptomatic and symptomatic COVID-19 patients. **(D)** Bar diagram representing the viral copy number of OP samples collected from patients with asymptomatic and symptomatic COVID-19 at different days of disease onset. **(E, F)** Graph depicting viral copy number of individual plasma and serum samples collected from asymptomatic and symptomatic COVID-19 patients. The Mann–Whitney (non-parametric, two-tailed) test was performed. All error bars were SD. p value ≤ 0.05 was considered statistically significant (*) and p value ≤ 0.01 was considered to be very significant (**).

The clinical and biological features of 12 hospitalized patients (asymptomatic and symptomatic) are presented in **Tables 1** and **2**. The viral load was assessed after their admission to the COVID hospital (**Table 3**). All the asymptomatic patients (nos. 1–6) recovered and were discharged from the hospital after 10–11 days. Two symptomatic patients (nos. 10 and 11) succumbed to COVID-19 during this period, whereas patient no. 9 passed away after getting discharged from the hospital. The asymptomatic patients (nos. 1–6) were identified as COVID-19 positive during contact tracing. The analysis of the biochemical data revealed abnormalities in few clinical markers such as CRP, LDH, serum glutamic-oxaloacetic transaminase (SGOT), serum glutamic pyruvic transaminase (SGPT), and ferritin. All the symptomatic patients had a higher level of CRP, and 50% of them displayed a high amount of serum LDH (~1.8–2.9-fold) than the normal range. Besides, they were detected with a higher percentage of serum SGPT (>2-fold) and serum SGOT (>2-fold), and almost 83% of the SARS-CoV-2 patients were detected with a higher level of ferritin than the normal range (**Tables 1** and **2**). These data suggest that patients with higher levels of the abovementioned biochemical parameters might experience severe pathophysiological complications after SARS-CoV-2 infection. For the treatment of this disease, all the patients were administered with pantoprazole (before food), azithromycin (once a day), ascorbic acid (2 h after food), and ivermectin (2 h after food), for a period of 10 days. Asymptomatic patients were discharged from the hospital on day 10. The symptomatic patients were found to have one or more comorbidities, especially type 2 diabetes (66.6%), indicating that other physiological conditions (**Tables 1** and **2**) are playing a major role in determining disease pathology. After the onset of the disease, samples were collected within 1–3, 5–7, and 8–10 days. In most of the asymptomatic (except one) and symptomatic patients (except two), the ΔΔCt values were increased in OP samples over time. However, there was no significant difference in the ΔΔCt values of asymptomatic and symptomatic patients (**Figure 1A**). This suggests that viral load in the OP sample does not correlate with disease severity. In contrast, the viral load estimation from plasma samples demonstrated that symptomatic patients had high viral load as compared with asymptomatic patients (**Figure 1B**). Three symptomatic patients (patient nos. 9, 10, and 11) who died of SARS-CoV-2 had a significantly higher amount of plasma viremia or viral load, compared with those who were discharged from the hospital. All the symptomatic patients (except one) were on ventilator support. The analysis of serum samples demonstrated that the ΔΔCt values of serum samples of asymptomatic patients were more than the symptomatic patients, and this finding correlated well with the ΔΔCt values of plasma samples for both asymptomatic and symptomatic patients (**Figure 1C**). Additionally, a similar pattern of ΔΔCt values for plasma and serum samples was observed for three more asymptomatic cases (data not shown). Hence, the result indicates that development of clinical complications is mostly associated to high viral load in plasma and serum samples. The viral copy number of OP samples was reduced over time in both asymptomatic and symptomatic patients, and there was no significant difference between the viral load of asymptomatic and symptomatic patients (**Figure 1D**). This suggests that both asymptomatic and symptomatic patients are capable of transmitting the disease equally. Plasma and serum samples of symptomatic patients had a high viral copy number than those of asymptomatic patients (**Figures 1E, F**). Therefore, disease severity is mostly linked with high viral copy number in plasma and serum samples.

**Table 1:** Demographic details and laboratory findings of SARS-CoV-2-infected hospitalized asymptomatic patients.

Characteristics	Patient 1	Patient 2	Patient 3	Patient 4	Patient 5	Patient 6
Age	47	70	29	51	40	29
Sex	M	M	M	M	M	M
Symptoms	Asy	Asy	Asy	Asy	Asy	Asy
Comorbidities	None	NA	None	NA	None	None
Travel history	Delhi	NA	Bangalore	None	Hyderabad	Hyderabad
Medication	Pantoprazole, azithromycin, ascorbic acid, and ivermectin	Pantoprazole, azithromycin, ascorbic acid, and ivermectin	Pantoprazole, azithromycin, ascorbic acid, and ivermectin	Pantoprazole, azithromycin, ascorbic acid, and ivermectin	Pantoprazole, azithromycin, ascorbic acid, and ivermectin	Pantoprazole, azithromycin, ascorbic acid, and ivermectin
Duration of stay in hospital	11 days	11 days	10 days	11 days	10 days	11 days
WBC count, 10^3^ cells per µl	3.9	5.9	5.8	05	8.7	5.8
Neutrophils, differential count (%)	43	43	50	66	71	55
Lymphocytes, differential count (%)	46	45	38	15	17	36
Monocytes, differential count (%)	08	09	09	13	09	07
Eosinophils, differential count (%)	03	03	03	06	03	02
Basophils, differential count (%)	0	0	0	0	0	0
RBC count, 10^6^ per µl	3.7	5.95	4.96	5.46	5.43	5.76
Hemoglobin, g/dl	10.4	13.6	15	14.5	14	17.4
MCV, fl/µm^3^	88.1	71.4	88.7	80.2	78.8	88.4
Platelet count, 10^3^ per µl	155	250	211	165	93	164
Ferritin, ng/ml	115	492	361	115	57	218
Serum bilirubin total, mg per dl	0.12	0.45	1.2	0.42	0.5	0.92
Serum SGOT, U/L	30	59	38	38	26	45
Serum SGPT, U/L	23	142	39	48	19	42
Serum alkaline phosphatase, U/L	48	91	89	64	94	127
Serum albumin, g/L	4.0	4.6	4.6	3.9	4.1	5
Serum globulin, g/L	2.7	2.7	2.5	2.3	2.4	2.7
Status of the patients	Recovered	Recovered	Recovered	Recovered	Recovered	Recovered

Asy, asymptomatic; NA, not available.

**Table 2:** Demographic details and laboratory findings of SARS-CoV-2-infected hospitalized symptomatic patients.

Characteristics	Patient 7	Patient 8	Patient 9	Patient10	Patient 11	Patient 12
Age	57	52	65	59	34	45
Sex	F	M	M	M	M	F
Symptoms	Fever, tiredness, headache, and joint pain	Dyspnea, myalgia	Dyspnea, fever, tiredness, myalgia	Dyspnea, myalgia	Dyspnea, myalgia	Dyspnea, myalgia
Comorbidities	None	Diabetic (type 2)	Diabetic (type 2)	NA	Diabetic (type 2)	Diabetic (type 2)
Travel history	Kolkata	NA	None	NA	NA	NA
Medication	Pantoprazole, azithromycin, ascorbic acid, and ivermectin	Pantoprazole, azithromycin, ascorbic acid, and ivermectin	Pantoprazole, azithromycin, ascorbic acid, and ivermectin	Pantoprazole, azithromycin, ascorbic acid, and ivermectin	Pantoprazole, azithromycin, ascorbic acid, and ivermectin	Pantoprazole, azithromycin, ascorbic acid, and ivermectin
Duration of stay in hospital	11 days	11 days	11 days	6 days	6 days	11 days
WBC count, 10^3^ cells per µl	9.5	5.6	11	3.5	6.3	5.3
Neutrophils, differential count (%)	43	83	91	55	77	66
Lymphocytes, differential count (%)	36	11	06	30	18	15
Monocytes, differential count (%)	10	05	03	08	05	13
Eosinophils, differential count (%)	11	01	00	07	00	06
Basophils, differential count (%)	00	00	00	00	00	00
RBC count, 10^6^ per µl	3.8	4.6	4.3	2.5	6.0	5.4
Hemoglobin, g/dl	11.5	13	12.7	8.6	13.7	14.5
MCV, fl/µm^3^	94.5	83.3	84.5	93.4	69.7	80.2
Platelet count, 10^3^ per µl	184	125	218	49	230	165
CRP, mg/L	95	60.12	52.38	18.93	NA	89
D dimer, µg/ml	NA	2.38	0.63	NA	NA	NA
Ferritin, ng/ml	105	1,000	492	968	NA	115
Serum LDH, U/L	NA	456.79	NA	724.33	NA	723
Serum bilirubin total, mg/dl	0.55	0.91	0.61	5.85	0.83	0.8
Serum SGOT, U/L	38	67	56	226	107	49
Serum SGPT, U/L	20	120	50	165	128	16
Serum alkaline phosphatase, U/L	89	134	34	61	85	100
Serum albumin, g/dl	4.0	3.5	3.3	2.2	4.5	4.0
Serum globulin, g/dl	3.1	2.7	2.7	3.6	3.2	3.1
Status of the patients	Recovered	Recovered	Demised	Demised	Demised	Recovered

NA, not available.

**Table 3:** Confirmation of COVID-19 by RT-PCR and virus isolation.

Patient no.	Month of admission	Nature of specimen	E gene (Ct values)	N gene (Ct values)	RNaseP gene (Ct values)	Virus adaptation
1	July, 2020	Oropharyngeal swab	17.5	18.6	32.2	No
2	July, 2020	Oropharyngeal swab	21.8	23.2	26.1	No
3	July, 2020	Oropharyngeal swab	19.5	20.8	26.9	No
4	July, 2020	Oropharyngeal swab	20.5	21.9	25.4	No
5	July, 2020	Oropharyngeal swab	19.1	20	25.2	No
6	July, 2020	Oropharyngeal swab	21.6	23.3	25.2	No
7	July, 2020	Oropharyngeal swab	19.4	22.9	25	Yes
8	July, 2020	Oropharyngeal swab	24.1	26.2	29.2	No
9	July, 2020	Oropharyngeal swab	16.4	18.7	25.7	No
10	July, 2020	Oropharyngeal swab	16.9	17.1	28.8	No
11	July, 2020	Oropharyngeal swab	17.5	18	26.9	No
12	July, 2020	Oropharyngeal swab	20.3	22.5	28.4	No

### Analysis of Antibody Responses Against SARS-CoV-2

**Figure 2:**
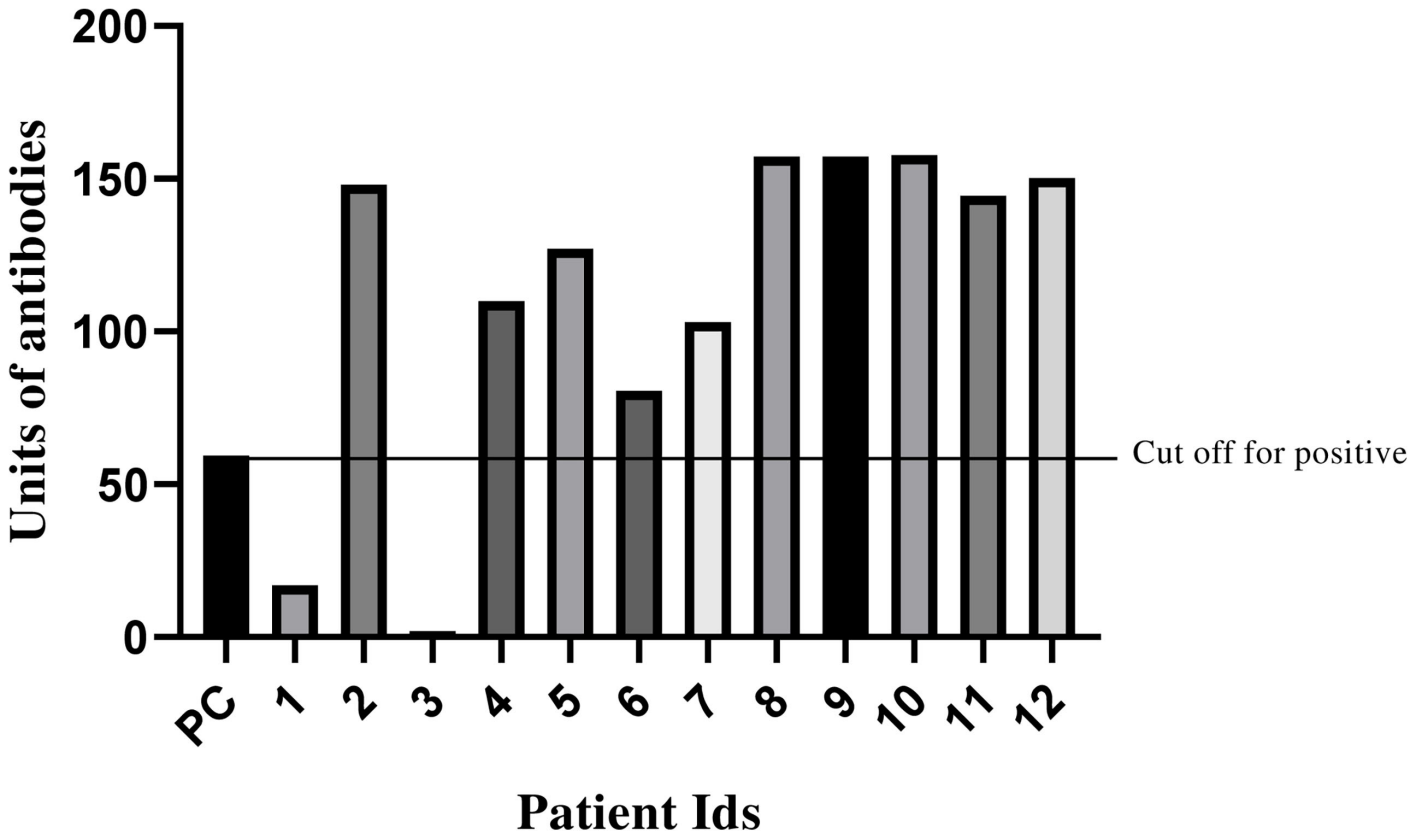
Analysis of SARS-CoV-2-specific (IgG+IgM+IgA) antibodies in asymptomatic and symptomatic patients. Bar graph representing antibody units (IgG+IgM+IgA) of plasma samples from a COVID-19 patient. Line depicting the cutoff value of positive samples which was 59.3. Negative (NC) and positive (PC) controls delivered by detection kit were included to warrant test validity.

To understand the antibody responses against SARS-CoV-2, virus-specific IgM, IgG, and IgA antibodies were estimated using plasma samples. Four out of six asymptomatic patients (patient nos. 2, 4, 5, and 6) along with all symptomatic patients were found to be ELISA positive (with low ΔΔCt values in the plasma) (**Figure 2**). The samples of three healthy individuals were collected as control and were negative for SARS-CoV-2-specific IgM, IgG, and IgA antibodies (data not shown). Thus, the data underline that patients with high viral titer developed a sufficient amount of antibodies. To comprehend infectivity, attempts were made to isolate the live virus from six clinical samples. However, one virus was successfully isolated from OP samples of patient 7 (viral load >10^7^ copies/ml), which has highest viral load among all the samples. This indicates the presence of a significantly high copy number of virus in this patient.

### Estimation of Circulating Cytokines and Chemokines in the SARS-CoV-2-Infected Asymptomatic and Symptomatic Patients

**Figure 3:**
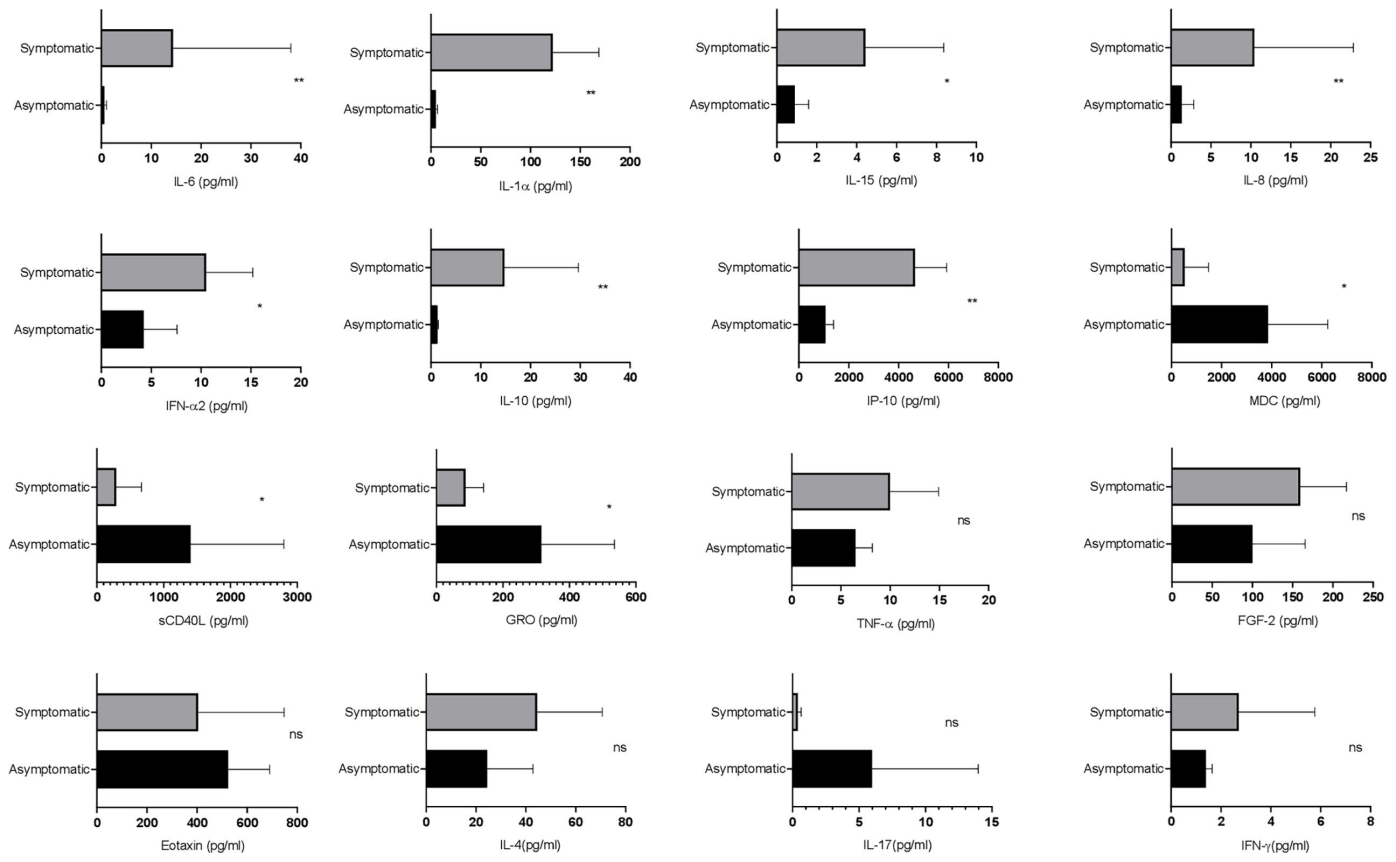
Evaluation of the cytokine responses between asymptomatic and symptomatic COVID-19 patients. The bar diagram depicting the expression levels of cytokines in pg/ml. Cytokines were measured after disease onset between asymptomatic and symptomatic patients. The Mann–Whitney (non-parametric, two-tailed) test was performed. *p < *0.05 was considered statistically significant (*), and *p < *0.005 was considered to be very significant (**). ns, not significant. All error bars were SD.

Next, the differential dynamics of circulating cytokines and chemokines in the SARS-CoV-2-infected asymptomatic and symptomatic patients were investigated. Interestingly, the levels of soluble CD40L, GRO, and MDC were higher in asymptomatic patients as compared with the symptomatic. On the other hand, the symptomatic patients showed higher levels of IL-6, IL-1α, IP-10, IL-8, IL-10, IFN-α2, and IL-15 in comparison with the asymptomatic patients (**Figure 3**), whereas there were no significant differences in the plasma levels of MCP-3, IL-1β, IL-17A, IL-12 p70, eotaxin, and TNF. Therefore, these data suggest that cytokines and chemokines may serve as “predictive indicators” of SARS-CoV-2 infection and contribute to understand the pathogenesis of COVID-19.

### Analysis of Total Circulating Antibody Isotypes

**Figure 4:**
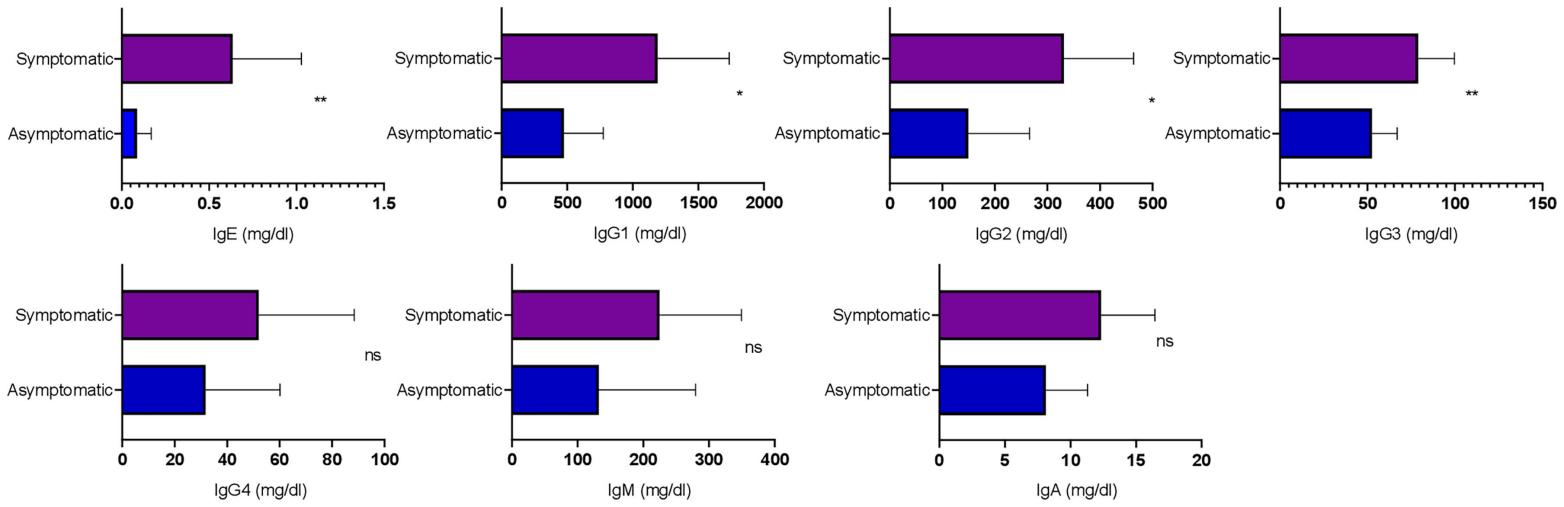
Analysis of the circulating antibodies in asymptomatic and symptomatic COVID-19 patients. The bar diagram representing circulating antibody isotypes (IgA, IgM, IgG1, IgG2, IgG3, IgG4, and IgE) which were evaluated from COVID-19-positive plasma samples. The Mann–Whitney (non-parametric, two-tailed) test was performed. *p <* 0.05 was considered statistically significant (*), and *p <* 0.005 was considered to be very significant (**). ns, not significant. All error bars were SD.

To understand the dynamics of circulating isotypes of antibody in the peripheral blood of SARS-CoV-2 patients, the Bio-Plex assay was carried out. There was no significant difference between IgA, IgM, and IgG4 in the two groups; however, IgE, IgG2, IgG3, and IgG1 were significantly higher in symptomatic than asymptomatic patients (**Figure 4**). This result indicates that inflammatory responses were high in these patients.

### Phylogenetic Analysis

**Figure 5:**
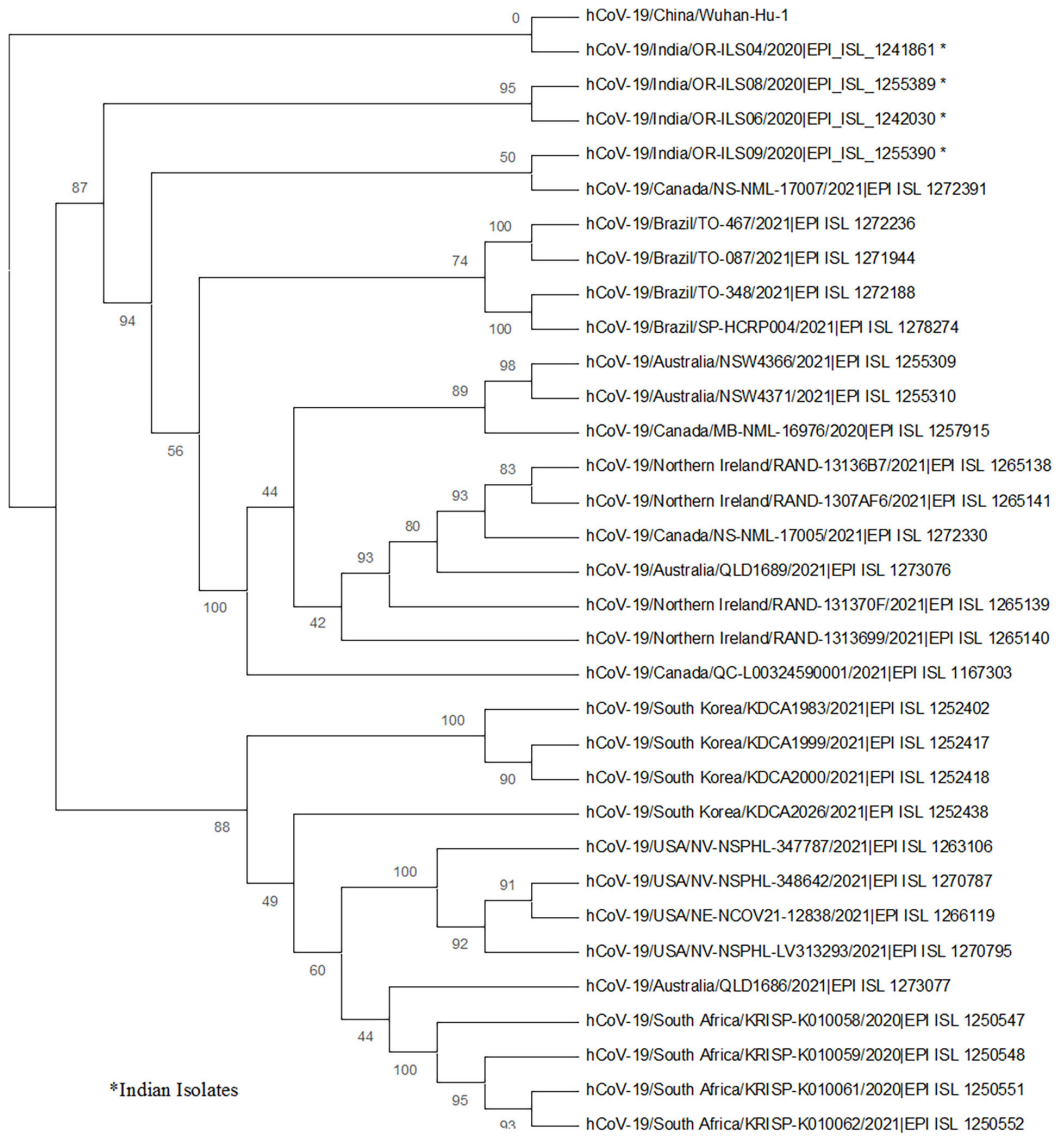
The phylogenetic analysis of the whole-genome sequences of four SARS-CoV-2 strains from human oropharyngeal swab samples is depicted. The phylogenetic tree was generated using the maximum likelihood method with 1,000 bootstrap value by the IQTREE2 tool. The tree was constructed using WH-01 (NC_045512.2) as a reference strain along with four strains from each of the countries, namely, USA, Brazil, Canada, Australia, South Africa, United Kingdom, and South Korea. The sequences for reference strains were retrieved from the GISAID database. The viral isolates are depicted by hCoV-19/country/strain ID/year of isolation/accession number. The bootstrap values are mentioned at major branch points of the tree.

In the current investigation, whole**-**genome sequencing was also performed for four SARS-CoV-2 strains (from one asymptomatic and three symptomatic patients). All four sequences were deposited in the GISAID database and the accession numbers are listed in **Table 4**. The phylogenetic tree was prepared using these isolates, and the strains from Wuhan, WH-01 (NC_045512.2); USA; Brazil; Canada; Australia; South Africa; United Kingdom; and South Korea were used as references. The phylogenetic analysis revealed the presence of three main clades: two (patient nos. 7 and 9) of the strains clustered to 20A clade, whereas the rest of the two (patient nos. 1 and 10) strains belonged to 19B and 20B clades (**Figure 5**).

**Table 4:** Accession IDs of GISAID submissions.

Patient no.	Virus name	Accession ID	Collection date	Location	Host	Passage	Sequencing technology	Assembly method
1	hCoV-19/India/OR-ILS04/2020	EPI_ISL_1241861	2020-07-01	Asia/India/Odisha	Human	Original	Illumina NextSeq 550	Reference based
7	hCoV-19/India/OR-ILS06/2020	EPI_ISL_1242030	2020-07-08	Asia/India/Odisha	Human	Passage 9	Illumina NextSeq 550	Reference based
9	hCoV-19/India/OR-ILS08/2020	EPI_ISL_1255389	2020-07-19	Asia/India/Odisha	Human	Original	Illumina NextSeq 550	Reference based
10	hCoV-19/India/OR-ILS09/2020	EPI_ISL_1255390	2020-07-21	Asia/India/Odisha	Human	Original	Illumina NextSeq 550	Reference based

### Mutational Analysis

**Figure 6:**
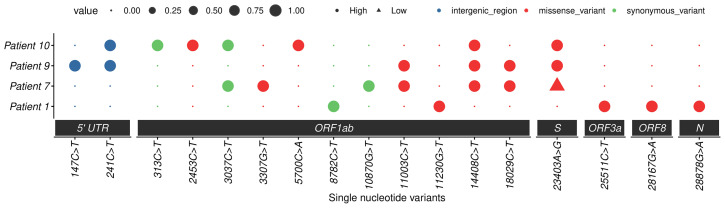
Mutation profiles of SARS-CoV-2 strains. Dot plot summarizing the single nucleotide variants in four studied cases. The *x*-axis represents the nucleotide change with position and region, and the *y*-axis represents cases. The dots represent the presence or absence of a mutation (small dot absent and large dot presence), and different colors represent different types of mutations. High-quality mutations (depth > 20) are represented by round dots and triangles represent low-quality mutations. PS: low-quality site only represented in spike 23403: A>G change.

Moreover, the sequence analysis revealed the presence of additional mutations, such as 13 in the Orf1ab region and 1 each in spike, Orf3a, Orf8, and nucleocapsid genes. All patients showed different combinations of mutations (approximately five to six) that are listed in **Table 5** and **Figure 6**. The Ref alleles/amino acid changes represent the genotype of Wuhan-Hu-01 strain and Alt alleles/amino acid changes represent the changes that occurred in Indian strains. Mutational analysis of amino acid unveiled a total of 11 missense mutations in the Orf1ab region, whereas 4 synonymous mutations were noticed in spike, nucleocapsid, Orf3a, and Orf8 regions. Interestingly, D614G mutation of the spike protein was observed in patient numbers 7, 9, and 10, and the analysis revealed that it is mostly linked with increased replication efficiencies and severe SARS-CoV-2 infection as the patients containing this mutation had high viral load and two patients succumb to death.

**Table 5:** Mutational analysis of the four SARS-CoV-2 strains compared with the Wuhan-Hu-1 (NC_045512.2) reference sequence.

Pos	Gene	Ref	Alt	AA Pos	Ref AA	Alt AA	Patient 1	Patient 7	Patient 9	Patient 10
147	*Orf1ab*	C	T	gp01 intergenic_region	–	–	0	0	1	0
241	*Orf1ab*	C	T	gp01 intergenic_region			0	0	1	1
313	*Orf1ab*	C	T	16 S	Leu	Leu	0	0	0	1
2453	*Orf1ab*	C	T	730 M	Leu	Phe	0	0	0	1
3037	*Orf1ab*	C	T	924 S	Phe	Phe	0	1	0	1
3307	*Orf1ab*	G	T	1014 M	Met	Ile	0	1	0	0
5700	*Orf1ab*	C	A	1812 M	Ala	Asp	0	0	0	1
8782	*Orf1ab*	C	T	2839 S	Ser	Ser	1	0	0	0
10870	*Orf1ab*	G	T	3535 S	Leu	Leu	0	1	0	0
11003	*Orf1ab*	C	T	3580 M	His	Tyr	0	1	1	0
11230	*Orf1ab*	G	T	3655 M	Met	Ile	1	0	0	0
14408	*Orf1ab*	C	T	4715 M	Pro	Leu	0	1	1	1
18029	*Orf1ab*	C	T	5922 M	Ale	Val	0	1	1	0
23403	*Spike*	A	G	614 M	Asp	Gly	0	1 (low depth)^a^	1	1
25511	*Orf3a*	C	T	40 M	Ser	Leu	1	0	0	0
28167	*Orf8*	G	A	92 M	Glu	Lys	1	0	0	0
28878	*Nucleocapsid*	G	A	202 M	Ser	Asn	1	0	0	0

a Low depth, dp <20.

## Discussion

A second wave of COVID-19 has grappled India, crossing 3.5 lakhs of new cases per day with an alarming death toll. An urgency persists to understand the clinical, epidemiological, immunological, and genomic characteristics of the virus for correlating with the pathophysiology of SARS-CoV-2.

The current investigation delineates and compares the clinical manifestations, laboratory findings, immunological responses, genome sequences, and treatment regimen of SARS-CoV-2 patients. Six asymptomatic and six symptomatic patients infected with this virus were studied for various parameters to understand the pathophysiology of this virus infection.

Patients with comorbidities displayed an increased severity in disease manifestation with some cases resulting in death. This observation was in accordance with a previous report where mortality as well as severity of the illness was increased for COVID-19-infected individuals with type 2diabetes as compared to non-diabetic patients (Kumar et al., 2020). This study further confirms that symptomatic patients had a higher level of CRP and ferritin than asymptomatic patients concurrent with previous reports (Velavan and Meyer, 2020; Yu et al., 2020). It was observed earlier that viral loads in nasal and throat swab samples were nearly the same in asymptomatic and symptomatic patients. The current study also confirms the above observation, suggesting that asymptomatic and symptomatic patients are equally capable of spreading the disease as there was no significant difference in viral load of both asymptomatic and symptomatic patients (Sohn et al., 2020; Zou et al., 2020). In contrast, the viral loads in plasma and serum samples were associated with clinical complications, which is consistent with other reports (Fajnzylber et al., 2020; Zhang et al., 2020). Furthermore, there might not be any threshold Ct value (viral load) responsible for clinical complications. Developing clinical complications may rely on other factors like comorbidities and cytokine profiles. A similar report by Abdulrahman et al. also suggests that just Ct values do not contribute in the disease severity or complications (Abdulrahman et al., 2021).

Additionally, the estimation of COVID-19-specific IgM, IgG, and IgA antibodies in SARS-CoV-2 patients indicated that patients with a high viral titer developed sufficient antibodies. A similar observation was reported in a study of patients from Hubei Province, China (Zhang et al., 2020).

In this study, only one strain (with >10^7^ copies/ml) could be isolated successfully. This demonstrates that the viral load was insufficient to successfully isolate the virus from other samples. A previous study also reported difficulty in the isolation of the virus when viral load is <10^6^ copies/ml (Wölfel et al., 2020). This explains the inability of isolating the virus from the remaining samples.

Symptomatic patients showed higher levels of IP-10, IL-8, IL-10, IFN-α2, IL-15, IL-6, and IL-1α, whereas the levels of soluble CD40L, GRO, and MDC were higher in asymptomatic patients. In addition, IL-10 and IL-6 have been associated with severe disease manifestations. On the other hand, IP-10 is considered to be a marker of ongoing inflammation, which in some cases predicts the mortality of the patients (Chi et al., 2020; Huang et al., 2020; Yang et al., 2020; Lev et al., 2021). Along with that, higher levels of IFN-α2, IL-15, IL-1α, and IL-8 in symptomatic patients indicate ongoing inflammation (Lucas et al., 2020; Qin et al., 2020). The levels of CD40L, GRO, and MDC were higher in asymptomatic patients, suggesting that they might have gone through immune suppression and repair phenomenon (Zaja-Milatovic and Richmond, 2008; Huang et al., 2012; Rapp et al., 2019). Previous reports have shown a significant difference in the expression levels of MCP-3, IL-1β, IL-17A, IL-12 p70, eotaxin, and TNF, while in the current study, there was no significant difference observed as small sample size was a limitation (Chi et al., 2020). A previous report by Ong et al. suggests that proinflammatory cytokines are increased only when the respiratory function is at the lowest level. In the current study, longitudinal profiling was not conducted; therefore, it was not very clear about the time point at which respiratory function was lowest with a significant increase of proinflammatory cytokines (Ong et al., 2020).

Symptomatic patients showed higher levels of IgE, IgG2, IgG3, and IgG1 serotypes, as compared with asymptomatic patients. IgE has already been reported to be high in COVID-19 patients, particularly those with type 2 diabetes. Out of six symptomatic patients in this study, four were suffering from type 2 diabetes and had enhanced IgE levels (Lucas et al., 2020; Zhao et al., 2020). An overall increase in IgG1, IgG2, and IgG3 responses in symptomatic patients explains the increase in inflammatory immune responses (Lucas et al., 2020). Since IgE was high in these patients, there was no significant difference in IgG4 level as they compete for fixation sites in basophils and mast cells (Kolfschoten et al., 2007). There was no difference in IgM and IgA levels of the two groups, suggesting that patients were at the early stage of infection, as the peak of virus-specific IgM is developed approximately 14–28 days after the onset of symptoms (Long et al., 2020).

The whole-genome sequencing and phylogenetic analysis revealed that the current isolates were clustered in 19B, 20A, and 20B clades; however, there were several additional unique mutations in different genes as compared with the Wuhan strain. Surprisingly, most of the changes were observed in the Orf1ab region. In India, these three clades were predominant in the months from May to July, 2020 (Maitra et al., 2020). It was observed in this study that the three symptomatic patients with high viral load, two of which had severe disease symptoms and died, were infected with the virus possessing the D614G mutation in the spike protein. This strengthens the previous hypothesis that infectivity rate and enhanced transmission of SARS-CoV-2 is associated with the D614G mutation (Raghav et al., 2020; Plante et al., 2021).

This study has indicated several interesting and significant outcomes, though with certain limitations. Primarily, all the clinical findings were correlated with viral RNA cycle threshold (ΔΔCt) value, which is capable of detecting dead virus particles also. Furthermore, the sample collection timing/interval was irregular and was solely dependent on the judgment of the physician and the condition of the patient. Finally, given the small sample size, the results must be interpreted with caution and validated in a larger cohort to fortify these conclusions.

In summary, this investigation highlights the ability of both asymptomatic and symptomatic patients to transmit the virus equally. This also demonstrated that the D614G mutation is mostly associated with higher virus replication efficiency and severe SARS-CoV-2 infection. These data also suggest that the enhanced levels of inflammatory markers such as CRP and ferritin can be predictive biomarkers for the critical condition of patients. Taken together, these results will contribute to a better understanding of the pathophysiology of SARS-CoV-2 infection and thereby advance the implementation of effective disease control strategies.

## Data Availability Statement

The datasets presented in this study can be found in online repositories. The names of the repository/repositories and accession number(s) can be found in the article/supplementary material.

## Ethics Statement

The studies involving human participants were reviewed and approved by the Institutional Human Ethics Committee, Institute of Life Sciences. The Institutional Ethics Committee (IEC)/ Institutional Review Board (IRB) reference number is 96/HEC/2020. Written consent informed to participate in this study was provided by the participants’ legal guardian/next of kin.

## Author Contributions

SoC and SaC designed the experiments. RS, SaC, SoS, ShS, SaS, GB, and AR collected the clinical specimens and demographic data. SaC, AD, and SK conducted the virological assays and RT-PCR. SoS and SaC performed the Bio-Plex and isotyping assays. SaC, AD, and SoS performed the ELISA experiment. AJ and EL prepared the samples for the whole-genome sequencing. SoC, SaC, and AD analyzed the virological data. SoS and SD analyzed the immunological data. AG, SR, and SW conducted the genomic data analysis. SoC, SaC, AD, SoS, and SP drafted the manuscript. AS, RD, SSe, TB, GS, PP, SR, SD, RS, SoC, and AP reviewed and edited the manuscript.

## Funding

This study was supported by core funding of the Institute of Life Sciences, Bhubaneswar, Dept. of Biotechnology, India. SaC and GB were funded by the CSIR fellowship and SoS was funded by the DBT fellowship.

## Conflict of Interest

The authors declare that the research was conducted in the absence of any commercial or financial relationships that could be construed as a potential conflict of interest.

## Publisher’s Note

All claims expressed in this article are solely those of the authors and do not necessarily represent those of their affiliated organizations, or those of the publisher, the editors and the reviewers. Any product that may be evaluated in this article, or claim that may be made by its manufacturer, is not guaranteed or endorsed by the publisher.
